# Exploring sociocultural factors and ubuntuism for women living with HIV in rural Zimbabwe

**DOI:** 10.4102/curationis.v47i2.2627

**Published:** 2024-11-13

**Authors:** Limkile Mpofu, Idah Moyo, Azwihangwisi H. Mavhandu-Mudzusi

**Affiliations:** 1 Department of Health Studies, College of Human Sciences, University of South Africa, Pretoria, South Africa; 2 Research Division, Jaylee Group, Pretoria, South Africa; 3 HIV Services, Population Solutions for Health, Harare, Zimbabawe; 4 School of Social Sciences, College of Human Sciences, University of South Africa, Pretoria, South Africa

**Keywords:** sociocultural factors, HIV-positive rural women, interpretive phenomenological analysis, patriarchy, stigma and discrimination, ubuntu

## Abstract

**Background:**

The focus of this study was on women living with HIV in rural Zimbabwe, who face many challenges, both in the family and community. Patriarchy compounds these problems as these women navigate access to health and other services.

**Objectives:**

The study sought to explore ubuntuism and the sociocultural factors that facilitate or constrain women living with HIV from accessing community services and resources.

**Method:**

An interpretive phenomenological analysis research design was utilised for the study. Purposive and quota sampling were applied to recruit 40 participants from six villages. Data were collected using in-depth interviews and focus group discussions that were audio recorded and transcribed. The data were analysed using the interpretive phenomenological analysis framework.

**Results:**

The study found that women living with HIV face stigma and discrimination in the form of social exclusion and reduced access to community-based health activities and empowerment opportunities. These challenges were compounded by the negative impact of patriarchy that gives rise to land, resettlement and acculturation challenges.

**Conclusion:**

Ubuntu is a reflection of mutual dependence and can be used to promote more compassionate relationships with those who are HIV positive in the community.

**Contribution:**

The study provides unique insights into the challenges experienced by women living with HIV and how ubuntu could mitigate those challenges so that it adds to the humanistic philosophies in interpersonal relationships.

## Introduction

According to the Joint United Nations Programme on HIV/AIDS (UNAIDS 2023), approximately 39 million (33.1–45.7 million) people were living with HIV at the end of 2022 globally. It is estimated that there are 1.2 million new HIV infections globally and of concern is the fact that 45% of all new HIV infections were among women and girls (all ages). In 2022, there were an estimated 1.9 million adolescent girls and young women (aged 15–24 years) living with HIV, compared with 1.2 million adolescent boys and young men (aged 15–24 years). With reference to sub-Saharan Africa, there were 210 000 new HIV infections among adolescent girls and young women (aged 15–24 years) in 2022. Women and girls (all ages) accounted for 63% of all new HIV infections (UNAIDS 2023).

The HIV incidence among women and girls has declined by 65% since 2010. Women, aged 15 years and over, however, still accounted for 61% of all people living with HIV in the region in 2022, with adolescent girls and young women aged 15–24 years, at an excessive risk of HIV infection (UNAIDS 2023). Of further concern is the fact that most adolescent girls and young women lack sufficient decision-making power about their sexual relations, contraceptive use and health care (Mpofu & Ganga-Limando 2024). In Zimbabwe, a total of 1.3 million were estimated to be living with HIV, while 17 000 were newly infected with HIV, with women contributing 50% of these new infections (Mpofu & Ganga-Limando 2024). In 2022, Zimbabwe had an HIV prevalence of 11% (UNAIDS 2023).

Patriarchy is characterised by gender inequalities, poverty and discrimination, which deny women and adolescent girls economic independence, as well as control over their sexual and reproductive health and rights (Leung et al. 2023; Mabaso et al. 2019). Patriarchy imposes restrictions on women, such as inheriting land and property, and limitations on their right to freedom of expression, association and movement. A cocktail of all these factors makes them more vulnerable to HIV. Sari et al. (2022) postulate that HIV-positive women are susceptible to pain, emotional abuse and possible rejection by significant others (life partners, relatives and friends). Evidence has shown that exposure to early and forced marriages, sexual violence and unwanted pregnancies among adolescents increases the risk of women contracting HIV (Ivanova, Rai & Kemigisha 2018) and being subjected to HIV stigma (Odimegwu, Akinyemi & Alabi 2017, 2018). In a study in Nigeria, Augustine Bala, Azman and Singh (2022) highlight that women were denied freedom of education and prevented from earning an income. The study emphasises how patriarchy further exacerbates the challenges of stigma and discrimination faced by women living with HIV.

Odhiambo et al. (2023) and Chapoto, Jayne and Mason (2011) observe that widows living with HIV are more likely to be further traumatised by accusations that they killed their husbands and, consequently, experience social isolation, reduced social support and property disinheritance.

The aforementioned reflects a serious departure from traditional African communalism as explicated in the African philosophy of ubuntu. ‘Ubuntu is a comprehensive phrase used by Africans to signify the attributes that embody the core human virtues of compassion and humanity’ (Rasweswe et al. 2024). According to Banda and Mudzanire (2019), the ubuntu philosophy emphasises respect and compassion for others. Muhammad-Lawal et al. (2023) posit that ubuntu is rooted in the community and has the following attributes: connectedness, humanness, reaching out, respect, kindness, warmth and understanding. Thus, the treatment of widows does not follow the fundamental values of ubuntu. This is a point also emphasised by Magezi and Khlopa (2021), for whom ubuntu is a normative value system that considers others as authentic beings, no matter their status.

Rasweswe et al. (2024) contend that ubuntu is acquired via socialisation in the community. The authors also view ubuntu as a universal African term that cannot be confined to one African language or community. This article, therefore, calls for a return to those values. When HIV-positive women are stigmatised (Leung et al. 2023), we see it as a departure from ubuntu, which, according to Dolamo (2013), is at the core of African ethics. Van Breda (2019) postulates that ubuntu is a representation of African beliefs and that a person’s identity is shaped by how they relate to other people. Practising ubuntu promotes an ethical environment because, as Mokhachane et al. (2023) put it, in ubuntu, ‘I am, because you are’ – an aphorism that should hold true no matter the circumstances. This research will explore the sociocultural factors that mediate, facilitate and constrain the health and empowerment of women living with HIV in the rural areas of Zimbabwe. It analyses the expression of ubuntu values in relation to the lived experiences of women living with HIV in a rural setting to extract the contribution of the ubuntu philosophy. The researchers collected data as part of a larger PhD research project, which looked at the model for mitigating stigma and discrimination against women living with HIV in rural Zimbabwe.

## Research methods and design

The researchers employed a phenomenological interpretative design. They used grand tour questions to allow the participants to freely account for their personal perspectives and experiences (Lentoor 2018; Van Manen 2014). This allowed for the identification of relevant behavioural outcomes, referents, cultural factors, facilitators and barriers for each particular behaviour of target population under investigation.

### Study setting

This study was conducted between July and August 2019, in Matabeleland South Province, Zimbabwe. Matabeleland South has the highest prevalence rate of HIV (17.6%) in Zimbabwe (UNAIDS 2023). This province also shares a homogeneous culture and language. Matabeleland South Province borders South Africa and Botswana. Because of South Africa and Botswana’s proximity to Matabeleland South, most of Matabeleland South’s population seek health care and employment from these neighbouring countries – South Africa and Botswana (David & Dube 2013). Besides, Matabeleland provinces are at an advantage as ‘the ethnic tribes share historical, kinship and linguistic ties with those from neighbouring countries’, making it easy for the male population to migrate and integrate with their new neighbourhoods (Sibanda & Khumalo 2017:6). This factor, while on the surface appears to be an advantage, has its disadvantage, as it facilitates the high HIV prevalence in Matabeleland South Province as noted in the statistics earlier.

### Sampling method

For this research, six villages in Matabeleland South Province were identified – one village per district. Then, through purposive and quota sampling, a total of 40 participants were selected from 75 households per village. This sample was extracted from the community health workers’ registers. In this village, it is a norm that anyone living with HIV will register under the community health worker so that they would access welfare that distinguishes between the deserving and undeserving and classifies most people living with HIV in the category of deserving the subsidy. However, no one is forced to register under these community healthcare workers. It is the circumstances that will force them to register, as one can only get assistance only if registered. The community healthcare workers are obliged to have a record of the assistance rendered to the people on their registers. The trend in this village is that each community health worker keeps a register of the people living with HIV under their care and the records of the care provided (Mpofu & Ganga-Limando 2024).

More so, purposive sampling was used to select the participants who could articulate their experience with eloquence to reveal the phenomenon of interest. This included adult woman (18 years and above), living with HIV and having tested HIV positive for at least 1 year from the date of the data collection. The women would also be living permanently in the village for at least 1 year after being tested HIV positive and be on antiretroviral therapy (ART) for at least 6 months. However, you would be excluded if you were women living with HIV, but on the treatment for any psychiatric condition and with less than a year being diagnosed with HIV, with no permanent residence in the area. Excluded from the study were also HIV-positive rural women not willing to participate in the study and those who were HIV negative. See also Table 1 on characteristics of the study participants.

**TABLE 1:** Characteristics of the study participants (*N* = 40).

Characteristics	*n*	%
**Age (years)**		
20–27	2	5
28–35	8	20
36–43	16	40
44–51	9	22.5
52–59	5	12.5
**Marital status**		
Single or divorced or widowed	26	65
Married	14	35
**Education**		
Primary or below	21	52.5
Secondary	12	30
Tertiary	7	17.5
**Employment**		
Unemployed or retired	27	67.5
Employed	13	32.5
**Time since HIV positive and ART initiation (years)**		
6+	12	30
4–6	21	52.5
0–3	7	17.5

*Source:* Adapted from Mpofu, L. & Ganga-Limando, M., 2024, ‘The lived experiences of HIV-positive women in rural Zimbabwe: A qualitative focus group study’, *South African Family Practice* 66(1), 1–8. https://doi.org/10.4102/safp.v66i1.5823

ART, antiretroviral therapy; HIV, human immunodeficiency virus.

All rural women living with HIV in the sampled villages were invited to participate, either in a focus group or individual interview – depending on where they resided. Three villages were too remote for the researchers; hence, the target group from these villages was recruited using convenience sampling for focus group discussions (FGDs), which made a total of 18 people, with each focus group consisting of 6 people.

The participants were later invited to their local clinics where the focus groups were conducted. The participants use these clinics to collect their antiretroviral treatment or attend their support group meetings. The target group from the other villages was considered for individual in-depth (ID) interviews.

### Data collection

The first author of this article conducted the individual face-to-face interviews and FGDs in English or Ndebele. However, most respondents preferred using English. We gathered information on sociocultural factors and ubuntuism in the questionnaire. This is because the researchers had a precise idea of the theoretical constructs they wanted to investigate as they pre-tested the instrument on two in-depth interviews to check their presumed connectedness. These 2 in-depth interviews were incorporated with the other 18 in-depth interviews to make them 20. An in-depth individual interview of approximately 50 min was conducted with each respondent, which was also digitally recorded, checked for quality and transcribed verbatim. On the other hand, the focus groups lasted an average of 90 min. The researchers discussed the findings within 24 h. The individual interviews and focus groups were triangulated with the researchers’ field notes, which were collected during the fieldwork. This would be included as data in this analysis.

**FIGURE 1:**
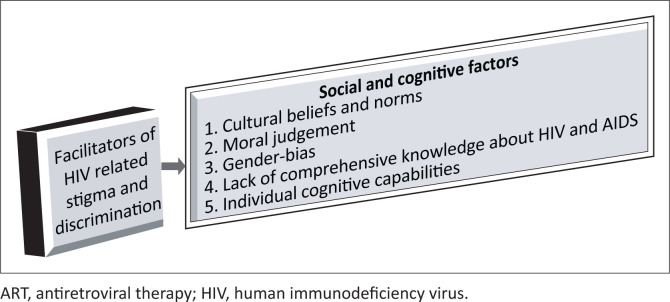
Extract from a framework for understanding being a woman living with HIV in rural Zimbabwe.

The following are the factors we considered, as extracted from our framework for understanding being a woman living with HIV in rural Zimbabwe (Figure 1).

Examples of the questions used to examine the participants’ experiences of being a rural woman living with HIV in Zimbabwe and how they respond to HIV-related stigma and discrimination are listed as follows:

1. Tell me about your life experiences as a woman living with HIV in this village.
2. What does it mean to you to be a woman living with HIV in this village?

The participants were further quizzed on their experiences of social prejudice and discrimination and on how it affected their psychosocial functioning and well-being.

The researchers’ field notes captured the interviewees’ body language and facial expressions.

### Data analysis

The researchers conducted an in-depth inductive qualitative analysis using the Interpretative Phenomenological Analysis (IPA) method. They used the following six main steps in analysing their qualitative data: familiarising oneself with the data, generating initial codes, searching for themes, reviewing, defining and naming and producing the report (Van Manen 2014). Their observation and comments were also recorded (Smith & Osborn 2015).

The authors identified related content across the data and allocated codes for their collected information to maintain confidentiality. Having also captured the non-verbal cues through field notes, the authors expanded on these notes to produce complete sentences and paragraphs, documenting what was observed and utilising the notes and audio recordings. Where Ndebele was used, the researchers translated it into English. The researchers read and reread the narratives and coded them to develop the themes and subthemes indicated in Table 2. This researcher utilised peer checking as a method of rigour. The mentor and the supervisor as experienced colleagues for this research re-analysed some of the data to ensure that the researcher has analysed the data correctly. ‘Peer checking reduced the effect of investigator bias and ensured that participants’ own meanings and perspectives were represented and not curtailed by the researchers’ own agenda and knowledge’ (Tong, Sainsbury & Craig 2007:356), in order to ensure trustiworthness. In summary, the researchers utilised the detailed transcription techniques, a schematic plan of systematic coding by means of computer programmes like Excel and NVIVO, as well as step-by-step counting in this qualitative research to ensure rigour and trustworthiness (Birth et al. 2016).

**TABLE 2:** Themes and subthemes.

Themes	Subthemes
Stigma and discrimination	Sense of social exclusion Reduced access to community-based health activities Reduced access to community-based empowerment opportunities
Patriarchy and its impact on HIV-positive women	Blame syndrome Land, resettlement and acculturation challenges HIV-positive women and educational opportunities

HIV, human immunodeficiency virus.

### Ethical considerations

The study observed all the ethical principles for research on human participants. Permission for the research was obtained from the University of South Africa Ethics Committee (reference no. HSHDC/847/2018) and the Medical Research Council of Zimbabwe (reference no. MRCZ/A/2398) before any data could be collected. The participants were informed about the consent process, that it was voluntary, that they could decline or discontinue participation at any time and that the interviews would be audio recorded. Participants were assured that data would be handled confidentially and that the results would be reported in a way that ensured anonymity. The researchers also ensured that pseudonyms were used as all identifiers were removed from the transcripts. Data were also stored in a password-protected computer. The researchers provided the study participants with a detailed information sheet that explained the study details. The participants gave an informed written consent after reading and understanding all the information.

## Results

Two themes and related subthemes emerged: stigma and discrimination and patriarchy and its impact on HIV-positive women (as shown in Table 2).

### Stigma and discrimination

The overarching theme relates to the stigma and discrimination experienced by HIV-positive women from significant others, as well as from the community. It has the following subthemes: sense of social exclusion, reduced access to community-based health activities and reduced access to community-based empowerment opportunities.

#### Sense of social exclusion

The subtheme describes how, after testing HIV-positive, the women felt socially excluded and experienced a loss of belonging. The following extracts capture that sense of exclusion:

‘We were taught to interact freely with others since our birth. This interaction is proof of our social identity as members of this community. It makes one feel that she is a member of the community with full rights, as recognised by our culture. But everything changed with our HIV-positive status. People, including your own close family members, distance themselves from you. It becomes difficult for people living with HIV, specifically for us women.’ (FDG 01, 49 years old, female, farm worker)

‘My in-laws told me to move out and set up my own homestead after my husband had died. I am socially excluded from them now. This is not fair at all, for me or my children.’ (IDI 16, 40 years old, female, agricultural worker)

For women living with HIV in rural Zimbabwe, the loss of social belonging meant exclusion from the existing social support system because they were denied of such use in their community:

‘Being a HIV-positive woman means everything terrible that you can think of. Before testing positive for HIV, we enjoyed everything in this community. We were recognised and considered full members of the family and the village. We received support from everybody in the family and community. We were invited to join all the social activities and play active roles. With the HIV-positive status, we no longer get support from our families and communities. Social support is critical in assisting you in building the confidence to conquer anything. But now … we are excluded from using all those resources. Yes, my sister [*referring to the researcher*], this is what it means to be a woman living with HIV in this community.’ (FDG 02, 32 years old, female, hairdresser)

‘I stopped my chicken business because of people’s behaviour toward me after testing HIV-positive. You know, I was breeding and selling chickens in my village. I was doing well in my business, but things changed after people became aware of my HIV status. Eish [*sighing*], this community is not supportive at all. They avoided me and my chickens. I had to stop my business as I didn’t get any buyers for my chickens.’ (IDI 13, 39 years old, female, entrepreneur)

#### Reduced access to community-based health activities

The subthemes highlight the meanings, beliefs and understandings women have about social and structural factors that facilitate and/or hinder their ability to achieve health and well-being:

‘You know, we need regular health check-ups as people living with HIV. The existing services are far away, but we have problems even accessing those services. We need a door-to-door medical check-up. The health workers/caregivers, who go around (sic), do not provide information on the importance of living healthy with HIV. They do not know how to deal with us without stigmatising us.’ (FDG 02, 53 years old, female, Creche teacher)

‘The community claims that they are holding us responsible for introducing HIV into the society and so they treat us poorly. We are harassed here and I fear them and sometimes I shy away from health care activities.’ (IDI 18, 38 years old, female, local boarding school cook)

#### Reduced access to community-based empowerment opportunities

Disclosing their HIV status meant that the women would be left out of economic empowerment training programmes, unlike before. This is confirmed in the following excerpts:

‘You know, we used to attend many trainings organised to empower women in the community. We applied the skills learned to achieve financial independence and fulfil our basic needs. We also applied the skills learned to establish our small businesses in the communities. Oh yes, we spend the income generated from these activities on food, health, and education. Now, with our HIV-positive status, we are no more [*sic*] invited to these trainings. They only invite us to trainings that talk about HIV and AIDS-related matters.’ (FDG 03, 41 years old, female, entrepreneur)

‘It’s a disgrace to be in this community because, once you are tested HIV-positive and being a girl, no one wants to spend money on your education. It is unfortunate for sure as some members of our families and communities, still consider HIV as a death sentence.’ (IDI 06, 27 years old, female, craftswork)

### Patriarchy and its impact on HIV-positive women

This theme contains the experiences of HIV-positive women in a patriarchal society and its impact. The theme comprises three subthemes: blame syndrome; land, resettlement and acculturation challenges and HIV-positive women and educational opportunities.

#### Blame syndrome

In a patriarchal society, there is the perception that it is the woman who brings HIV into the marital relationship. This view is demonstrated in the following extracts:

‘When my husband passed on, because of HIV and AIDS, I was pained by how I was treated by my mother-in-law. I was accused of having brought HIV into the marriage. She blames me for being responsible for the death of her son. She even told my children I killed my husband, because of my uncaring and defiant behaviour.’ (IDI 04, 36 years old, female, teacher)

‘My husband divorced me after I tested HIV-positive. He refused to go for an HIV test himself. I tried everything to keep him, but I failed. He indicated that he could not live with an HIV-positive woman.’ (IDI 20, 39 years old, female, entrepreneur)

#### Land, resettlement and acculturation challenges

HIV-positive women experience stress that emerges from their diagnosis. Such stress takes a ding on women’s health as they try to come to terms with living with HIV in a society that is discriminatory. In a patriarchal society, land, land ownership and/or access to land is often associated with the man and his male children. When he dies, the widow is at the mercy of his male relatives – more so if she has no male child. It means that the women living with HIV have no land rights, which affects their financial freedom, as they would have to depend on their male relatives. This is what came out from one of the FGDs:

‘You know, when your spouse dies of HIV and AIDS-related disease, you, as the widow, have nothing in your name. In my situation, I have no sons. According to the community, the number of years I lived with the deceased is immaterial. I was stripped of our land and decisions related to it.’ (FDG 02, 50 years old, female, farm worker)

#### HIV-positive women and educational opportunities

This subtheme reflects another negative aspect of patriarchy with respect to women and education. The following excerpt is about the experiences of a female university student after testing HIV positive:

‘I was in my first year of university when I was tested HIV-positive. I shared the news with my legal guardian, who was my support system and responsible for my university fees. His response was shocking, I was told that I should discontinue my studies, as he could not continue paying fees for an HIV-positive woman. I ended up in a forced marriage. The man was older but perceived to be rich. This, I was told, would change our family’s economic fortunes.’ (IDI 03, 22 years old, female, maid)

What also emerged from one of the FGDs was how being HIV positive as a young woman can further exacerbate existing educational and economic challenges that young women face in a patriarchal society. This was expounded in the following extracts:

‘You know the role of education for women in this country. The economic gains of educating a girl are immense. However, when you test HIV-positive as a young woman, you are doomed. No one wants to pay for your education or provide some support once they are aware of your HIV-positive status. It is sad. For some members of our families and communities, HIV is still a death sentence.’ (FDG 01, 29 years old, female, farm worker)

‘Look at me, I quit education not because I wanted to. Its because I got sick and tested HIV-positive. No one ever wanted to waste their money on me, a girl who could die any time. At at least that’s what they thought, but I am still here. Not educated. I am only 25 years imagine.’ (IDI 19, 25 years old, female, unemployed)

## Discussion

Ubuntu philosophy speaks about the interconnectedness of humans. It further promotes the importance of the community affirming their own humanity by recognising the humanity of others, relating humanely and working harmoniously and cooperatively with others as brothers and sisters (Rasweswe et al. 2024). In this study, the researchers found that HIV-positive rural women experienced challenges in social and cultural adjustments, which were exacerbated by conflicting cultural values, physical health problems, HIV-related stigma and discrimination, poverty and isolation. Social exclusion meant that the women living with HIV felt isolated and experienced a loss of belonging. Isolation, in turn, led to depression. These findings concur with those by Charles et al. (2012), who claim that depression can lead to either the perception of having a smaller support network or interactions with fewer network members. While it is true that the African extended family system is an integral part of the ubuntu philosophy, in this study, it was revealed that the women were often forcibly removed from their land. This is because, despite the changes in customary law, the woman is often regarded as a minor in rural areas. They are often regarded as being under the guardianship of their husband and incapable of owning property. This increases their risk of experiencing psychological problems and post-traumatic stress disorder. Such treatment would indicate that communities are drifting away from the ubuntu philosophy (Chapoto et al. 2011; Odhiambo et al. 2023). The community failed to contextualise, assimilate and localise the concept of inclusive citizenship by discriminating against the women living with HIV, thereby deviating from values subscribed by the African ubuntu philosophy. In this study, the extended family system did not use ubuntu as Magezi and Khlopa (2021) advocated. Because of the backwardness in rural areas, many women were frequently disconnected from their material possessions, particularly land, or separated from family members because of domestic violence, abuse and divorce. They were also subjected to intermittent disruption to access to health services and other challenges related to isolation or integration into the society.

The findings of this study were that HIV-positive widows, especially those with no male children found themselves in a precarious situation with regard to the land they had tilled with their husbands. Often they were blamed for killing their husbands and thus did not deserve land. Contrary to the findings here studies elsewhere attached to the blame of HIV infection on the male spouses (Chibango & Potgiter 2023). This is further corroborated by studies by Madiba and Ngwenya (2017).

With regard to women and land tenure, the United Nations also notes how, in keeping with partriachy, it is the male relative who assumes control of the land after the male spouse passes away (UN Women 2018). In this study, as also observed by the United Nations, the women found themselves stripped of their land when their spouses passed on and accused of having killed them. This goes against ubuntu philosophy, according to which vulnerable members of society like widows and children are protected (eds. Mukuni & Tlou 2021).

Partriachial societies often attach blame on women when things go wrong. In this study, women were sometimes accused of having brought HIV into their marriage. This is consistent with studies by Peacock (2013) where he found that women were implicated in having brought HIV into the marriage while their male counterparts were exonerated. This was inspite of the multiple partners they had, which was often equated to normative masculinity.

However, ubuntu philosophy is not about buck passing but is reconciliatory in its approach to human relationships (Odhiambo et al. 2023).

According to UNAIDS (2023), in sub-Saharan Africa, new HIV infections are higher among adolescents and young women. The finding of this study was that one of the participants was forced to terminate her university studies because her guardian did not want to pay fees for someone HIV positive. This is despite the fact that UNAIDS advocates for greater investment in education for adolescent girls and young women as an empowerment strategy for females. In the same vein, Bago et al. (2021) call for policymakers to craft policies that invest in promoting girls’ education to reduce the HIV prevalence among women. The ubuntu philosophy if adopted would not see adolescent girls and young women drop out of school because of lack of funding as a result of her HIV status. This is because underpinning ubuntu philosophy is the belief that a child belongs to the entire community and so is its welfare (Olaore & Drolet 2016). Also, worth noting is the postulation by Mugumbate and Chereni (2019) that ubuntu if adopted would create a compassionate environment ideal for child rearing.

Our findings further indicated that these women also faced HIV-related stigma challenges that could undermine their health because of stress related to having to adapt to a new life that included living with HIV or discriminatory treatment. Similar to the research findings by Leung et al. (2023) and Mabaso et al. (2019), these women were subjected to disproportionate exposure and vulnerability to adversities and multiple health risks. Because these are women living with HIV, they are more vulnerable to stressors, and they, therefore, need social support and resources to manage their health. The only redeeming factor for a better quality of life would be to apply the principles of Ubuntu philosophy in full. It is true that the quality of life is influenced by different social factors, including relationships, friends and the community (Alsubaie et al. 2019; Sari et al. 2022). Our study, therefore, notes that social support from friends or significant others would improve the quality of life in social relationships in cases similar to this study. In their research, Charles et al. (2012) found that when comparing family social support and social relationships, family social support increased the quality of psychological well-being of the individual. This was visible where individuals looked for emotional support from their family, especially in times of critical crises as in testing HIV positive. Again, social support from significant others is also a predictor of social relationships and, as such, is enhanced by ubuntu (Van Breda 2019). In this study, we saw dwindling community interactions because of the stigmatisation of women living with HIV. We are concerned about whether there is a gradual erosion of humanness in health care. It is crucial that the ubuntu philosophy be passed on to the next generation.

The sociocultural contexts within which these women live and how they navigate life when it is determined that they are living with HIV/AIDS are fundamental to their ability to access the requisite goods (land, financial freedom, economic independence and even education) and services, as well as relationships. The roots of ubuntu lie in the humanist African philosophy, where the idea of community is one of the building blocks of society (Praeg 2017). The most striking feature of the traditional culture of different African nations is their non-individualistic character, where community forms the cornerstone of African thought and life (Nicolaides 2022). In the Zimbabwean culture, the ubuntu philosophy is embedded in the socialisation process. According to Samkange (1980), Zimbabwean children are taught to embrace the values of ubuntu by their parents or guardians, which encompass, among others, the values of integrity, wisdom, hard work, economic productivity and social solidarity – as people or relationships are part of their identity as humans.

## Conclusion

The lesson drawn from the accounts of these rural women living with HIV is that ubuntu promotes a strong sense of mutual dependence in the community. As a way of life, ubuntu should give direction and alternative approaches to building human relations and effective intercultural communication (Mukuni & Tlou 2021). While we concentrated on a particular sample of rural women living with HIV, our findings point towards a range of factors, pathways and strategies that may be instrumental in shaping the health of other marginalised women or the magnitude of adverse health effects from various stressors. As people live in cross-cultural contexts, there is a need to understand and appreciate the perspectives and worldviews of others in order to shape our understanding of humanity, upbringing and engagement in social environments. The starting point would be to investigate indigenous philosophies of life, such as ubuntu, and to examine how such value systems can survive alongside opposing or similar traditions. This research aimed to improve the understanding of cultural values by discussing the central tenets of the African philosophy of ubuntu. The ubuntu philosophy teaches us the importance of preserving harmony, being pleasant and sensitive in those relationships for the welfare of other people. In our African culture, the well-being of others, such as the family, clan or society, is viewed as crucial as that of the self.
